# Patterns of Parenting and their Associations with Bereaved Children’s Maladaptive and Adaptive Functioning

**DOI:** 10.1007/s10802-025-01403-0

**Published:** 2026-02-18

**Authors:** Taylor R. Napier, Amanda J. Hasselle, Charis J. Stanek, Mia Chambers, Kathryn H. Howell

**Affiliations:** 1 Department of Psychiatry and Behavioral Health, Nationwide Children’s Hospital, 444 Butterfly Gardens Dr, Floor 4, Columbus, OH 43215 USA; 2 Department of Psychology, Rhodes College, Memphis, TN 38112 USA; 3 Department of Social Work, The Ohio State University, Columbus, OH 43210 USA; 4 Department of Psychology, The University of Memphis, Memphis, TN 38152 USA; 5 Department of Psychology, University of Wisconsin-Madison, Madison, WI 53706 USA

**Keywords:** Bereavement, Parent-child relationship, Posttraumatic stress, Psychosocial difficulties, Posttraumatic growth, Resilience

## Abstract

The parent-child relationship influences children’s mental health (e.g., posttraumatic stress symptoms, psychosocial difficulties) and adaptive outcomes (e.g., posttraumatic growth, resilience) following the death of a loved one. The current study employed a person-centered approach to identify patterns of parenting and their associations with bereaved children’s functioning. This study included 112 bereaved children, aged 8–17 (*M*_*age*_ = 12.41, *SD* = 2.58; 52.2% female), recruited from community organizations in the MidSouth, United States. A latent profile analysis was used to identify parenting subgroups and examine differences between classes on children’s posttraumatic stress symptoms, psychosocial difficulties, posttraumatic growth, and resilience. A three-class solution best fit the data: Passive Parenting (42.86%), Negative Parenting (34.82%), and Positive Parenting (22.32%). Children in the Positive Parenting class reported fewer psychosocial difficulties than those in the Passive (Est. = −8.33, *p* < .001, *d* = 0.86) and Negative (Est. = −15.82, *p* < .001, *d* = 1.67) Parenting classes; those in the Passive Parenting class had fewer psychosocial difficulties than the Negative Parenting class (Est. = −7.49, *p* < .001, *d* = 0.79). Children in the Positive Parenting class reported higher levels of resilience compared to the Negative (Est. = 8.95, *p* = .015; *d* = 0.51) and Passive (Est. = 11.44, *p* < .001; *d* = 0.69) Parenting classes; no significant differences on posttraumatic stress symptoms or posttraumatic growth were found. Bereaved children whose parents are more involved, prioritize open communication, and use more positive reinforcement strategies report fewer difficulties and more resilience. In contrast, children’s trauma-related outcomes following a death were not influenced by parenting patterns and may be better explained by other relational factors.

**Supplementary Information:**

The online version contains supplementary material available at 10.1007/s10802-025-01403-0.

## Patterns of Parenting and their Associations with Bereaved Children’s Maladaptive and Adaptive Functioning

In the aftermath of the death of a loved one, children exhibit diverse grief reactions with some reporting heightened challenges including posttraumatic stress symptoms (PTSS; Spuij, Reitz, et al., 2012) and psychosocial difficulties (i.e., social, emotional, and behavioral challenges; Salloum et al., 2019), while others show high levels of posttraumatic growth (PTG; Wolchik et al., 2009) and resilience (Lin et al., 2004). The parent-child relationship, cultivated via engaged parenting practices and supportive interactions, constitutes a critical relational factor that can influence children’s functioning following a death (Alvis et al., 2023; Wolchik et al., 2009). The importance of the parent-child relationship in helping children navigate grief is commonly recognized, but few studies have explored enacted parenting practices among bereaved families (Alvis et al., 2023). Most of the available research assesses how one specific facet of parenting (e.g., maternal warmth, positive parenting) is associated with singular child outcomes (e.g., PTSS; depression; Morris et al., 2016; Williamson et al., 2017); however, parent-child relationships are complex and multifaceted, so the use of a person-centered approach could help elucidate meaningful parenting patterns that are differentially related to bereaved children’s well-being. Guided by the Relational Development Systems Metatheory (Lerner et al., 2018) and the Parent Development Theory (Mowder, 2005), this study explored how child-reported profiles of parents’ Involvement, Positive Parenting, Deficient Monitoring, Ineffective Discipline, Open Communication, and Problems in Communication were associated with their maladaptive (i.e., PTSS, psychosocial difficulties) and adaptive (i.e., PTG, resilience) functioning after the death of a loved one.

### Childhood Bereavement and Children’s Functioning

Childhood bereavement refers to the experience of losing a loved one before the age of 18 and it is typically followed by a grief reaction, or the emotional experience that arises after a death (Melhem et al., 2011). Grief reactions range from “normative” or expected grief (e.g., sadness, anger, appetite changes) to complicated grief (Melhem et al., 2011). Complicated grief reactions are characterized by an intense yearning for the deceased, negative beliefs about the meaning of death that contribute to difficulty accepting the loss, avoidance of reminders about the death, and trouble integrating the loss into their life (Nader & Salloum, 2011). Approximately 10% of bereaved children exhibit complicated grief reactions (Melhem et al., 2011).

Complicated grief reactions can heighten the risk for other psychological challenges, including elevated PTSS and other emotional and behavioral difficulties (e.g., depression; Bui, 2018). PTSS in bereaved children includes symptoms associated with functional impairment such as intrusive thoughts, flashbacks, and avoiding reminders of the death (Spuij, Reitz, et al., 2012). Previous research shows that nearly 7% of children experience PTSS following the death of a loved one (Melhem et al., 2013). Psychosocial difficulties are also common after the death of a loved one (Salloum et al., 2019) and may endure for years (Rosenberg et al., 2015). Research demonstrates that children who experience loss are three times more likely to show internalizing problems (e.g., depressive symptoms) compared to their nonbereaved peers (Melhem et al., 2008), while others exhibit heightened externalizing problems including aggression, risky decision making, and substance use (Howard Sharp et al., 2020; Rosenberg et al., 2015).

The impairing effects of grief on children’s mental health have largely dominated the literature, yet many bereaved children display positive adaptation including posttraumatic growth (PTG; McClatchey, 2020; Salloum et al., 2019). PTG reflects an individual’s perception of positive changes that occur as a result of experiencing adversity (e.g., better relationships, optimistic outlook on life; Tedeschi & Calhoun, 1996). Children’s experiences of PTG following a death are understudied compared to their adult counterparts (Kilmer et al., 2009). A small body of literature has identified PTG themes from children’s reports that include increased maturity, empathy, and altruism (Kilmer & Gil-Rivas, 2010; Salloum et al., 2019). Resilience constitutes another adaptive outcome that is underexplored in the bereavement literature despite evidence that a large proportion of children show resilience in the aftermath of a loss (44%; Lin et al., 2004). Definitions of resilience vary across studies but generally refer to an individual’s ability to overcome and thrive in the face of adversity. In the current study, resilience is defined as personal qualities and skills (e.g., adaptability, optimism, perspective-taking, resourcefulness, self-efficacy) that can be harnessed to effectively cope with distress (Connor & Davidson, 2003).

### Theoretical Framework: Parent-Child Relationships in the Context of Bereavement

Children’s maladaptive and adaptive functioning following a death are heavily influenced by the caregiving context (Lerner et al., 2018). The Relational Developmental Systems (RDS) Metatheory posits that children’s adjustment to adverse experiences is contingent upon.

their support resources at home and in their surrounding environments (e.g., school, neighborhood; Lerner et al., 2018). Children’s developmental needs at the time of the adversity also impact the manifestation and intensity of symptoms (Overton, 2015). Regarding bereavement, the RDS Metatheory suggests that positive adjustment following a death is influenced by the closeness of the fit between the child’s emotional/developmental needs and the capacity for their surrounding environment, including their caregivers, to meet those needs (Lerner et al., 2018; Nader & Layne, 2009). Importantly, parent-child relationships are reciprocal such that children who are willing to ask for help from their caregivers may receive more support while at the same time parents who are more in tune with their children’s needs may offer support before being asked. The Parent Development Theory (PDT; Mowder, 2005) complements the RDS Metatheory as it provides a theoretical framing of parents’ on-going ability to adjust their perspectives on parenting and their parenting behavior. The core tenants of PDT center on parents’ views of bonding with their child, use of discipline, transmission of knowledge, protection, and correctly identifying and responding to their child’s needs. These parental roles are shaped by a parent’s own characteristics and experiences as well as their child’s characteristics and experiences, including the death of a loved one. Taken together, there is likely considerable variation in how children view their interactions with their parents; these perspectives are informed by their parents’ pre-existing parenting style, the developmental and grief-related needs of a child, and the parent’s ability to adapt and respond to those needs.

### Parent-Child Interactions and Bereaved Children’s Functioning

The broader parenting literature documents the association between positive parenting practices and reduced PTSS (Afzal et al., 2023; Williamson et al., 2017), as well as fewer internalizing and externalizing problems (Bøe et al., 2014), among children. These studies suggest that parental support, empathy, and emotional availability are linked with improved mental health (Afzal et al., 2023; Williamson et al., 2017), but little work has examined the association between parenting practices, PTSS and psychosocial difficulties specifically in bereaved children. One study with a small sample of children who lost a sibling (*n* = 62) showed that positive parenting and parental involvement were significantly correlated with lower PTSS and fewer symptoms of depression (Morris et al., 2016). Another study that evaluated the associations between maternal communication, maternal depressive symptoms, and children’s depressive and maladaptive grief symptoms among 38 parentally bereaved children found that mothers who exhibited more warm, responsive, and engaged communication had children with fewer mood challenges and less complicated grief reactions (Shapiro et al., 2014). Regarding more problematic aspects of parenting, research shows that negative parenting strategies (e.g., avoiding conversation about the trauma, overprotection, harsh parenting) are linked with greater PTSS symptoms (Williamson et al., 2017) and poorer psychosocial outcomes in children (Bøe et al., 2014). Among children who experienced the death of a sibling, problematic mother-child communication was tied to higher levels of externalizing problems (Howard Sharp et al., 2020). These studies offer valuable information on parenting and bereaved children’s mental health, but most studies explored only one specific parenting variable, and their small samples prohibited the use of advanced statistical analyses.

Research is even more limited on positive parenting variables and children’s PTG and resilience. A recent review of PTG in parentally bereaved children highlights the conceptual importance of caregivers in supporting their child’s adaptive grief process through strategies such as positive parenting, being sensitive to children’s needs, having open and honest conversations, and facilitating connection to the deceased (Şimşek Arslan et al., 2022); however, these parenting strategies have yet to be examined empirically in this population. One study showed that parental engagement in grief services predicted greater child resilience (i.e., stronger locus of control), which was linked to fewer complicated grief symptoms; specific parenting behaviors enacted at home were not assessed (Cipriano et al., 2023). In addition, behavioral management strategies such as consistent discipline are also associated with improved mental health in children exposed to adversity (Short et al., 2023), though explicit associations between discipline strategies and children’s adaptive and maladaptive outcomes after a death remain understudied. This study aimed to fill a significant research gap by examining the associations between positive and negative parenting practices and children’s adaptive and maladaptive outcomes following the death of a loved one.

In line with Parent Development Theory (Mowder, 2005), parenting in the context of bereavement may be particularly difficult due to new life stressors and challenges. For example, some parents are now single parenting, which can increase their caregiver burden while they simultaneously cope with their own grief (Jasmita et al., 2024). During this time, parents may have gaps in social support or choose to disengage from their social networks (McClatchey, 2020). These changes to parents’ relationships and subsequent feelings of loneliness could negatively impact relationships with their children (Ellis et al., 2013). Further, parents may take on additional household tasks, new logistical and financial responsibilities, and more emotional demands as they support their grieving child. These stressors can diminish their parenting quality and ultimately their child’s mental health (Saldinger et al., 2004). More specifically, parents’ communication skills, warmth and involvement, and use of appropriate discipline can all be compromised in the aftermath of a death, which can affect children’s functioning (Bergman et al., 2017). Notably, most literature examining parenting in bereaved samples explores associations between broadband measures of positive or negative parenting and children’s functioning. This approach is limited as it does not fully capture the variation in parenting behaviors across domains (e.g., use of praise, discipline, monitoring, communication).

### Identifying Distinct Parenting Patterns

Person-centered approaches, such as latent profile analysis, can detect underlying patterns across a set of variables and highlight subgroups of respondents with similar responses (Saldinger et al., 2004; Von Eye & Wiedermann, 2015). Prior person-centered work exploring patterns of parenting and associations with child outcomes has not been published with bereaved samples despite past research highlighting the potential negative effects of bereavement on the family system. Indeed, the new emotional and logistical demands associated with the death of a loved one could uniquely influence the parent-child relationship. For some families, parents may become more involved or engaged with their child to ensure their child is fairing well after the death. In contrast, some caregivers may have significant difficulties attending to their child’s emotional needs when they are managing additional financial and household burdens. In a study that used a person-centered approach with a non-bereaved sample, results showed three classes of parenting (i.e., positive, negative, intermittent) among mothers of young children (aged 3–5); children in the negative class had more physically aggressive behaviors compared to the positive parenting class (Tzoumakis et al., 2015). These findings were similar to Borden et al. (2014), who conducted a study with mothers of children (aged 3–8) with behavioral challenges, except they identified four classes of parenting, including a “neither positive nor negative” category (Borden et al., 2014). Another study identified two latent parenting profiles (i.e., High-Risk and Low-Risk) in a sample of families enrolled in a parenting program for children (Weeland et al., 2023). Notably, these profiles were derived from parenting behaviors (e.g., praise and harsh discipline) as well as parent (e.g., education, age, mental health) and child (e.g., hyperactivity, emotional problems) risk factors. Children in the High-risk class had more emotional and behavioral difficulties and their caregivers were less educated and younger compared to the Low-risk class; praise and harsh discipline were similar across classes (Weeland et al., 2023). The variability in profile identification across past research illustrates the complexities of parent-child interactions that may be influenced by contextual or environmental factors. Moreover, these studies used samples of young children with behavioral problems and their mothers. To date, there are no known studies that employ a latent profile analysis to examine parenting behaviors from the perspective of bereaved children and how those profiles relate to their maladaptive and adaptive functioning. Such work is critically needed to illustrate how children with similar perceptions of parenting view their own mental health and well-being.

### Loss-Specific Variables and Demographic Factors

Loss-related factors, such as time since death and relationship to the deceased, can also contribute to children’s functioning (Haine et al., 2008). Some work suggests that initial reactions of sadness and dysphoria following a death can result in long-term mental health complications (Haine et al., 2008), while other studies indicate that complicated grief reactions lasting longer than nine months are linked to more severe mental health problems (Melhem et al., 2013). The type of death children experience may have unique effects on the parent-child relationship, such that those who lose a parent may undergo significant changes to their interactions with the surviving parent (Wray et al., 2022). Finally, child age is an important demographic variable to consider when examining the parent-child relationship as research highlights the changes in the parent-child dynamic as children age and develop more autonomy and independence (Dowdney, 2008).

## The Current Study

Bereaved children exhibit different trajectories of functioning following a death that are largely influenced by the parent-child relationship (Alvis et al., 2023; Wolchik et al., 2009). Very few studies have examined the role of parenting practices in shaping children’s maladaptive (i.e., PTSS, psychosocial difficulties) and adaptive (i.e., PTG, resilience) outcomes in the wake of loss. Guided by the RDS Metatheory (Lerner et al., 2018) and the PDT (Mowder, 2005), the current study adds to the literature by implementing a person-centered approach to uncover parenting patterns using both positive and negative parenting indicators among children who have experienced the death of a loved one. Previous studies using only parenting variables as profile indicators among non-bereaved samples suggest that at least a 3-profile solution will emerge (Borden et al., 2014; Tzoumakis et al., 2015), with distinct classes capturing more optimal parenting strategies, more negative parenting strategies, and a mixture of parenting practices. It was hypothesized that 1) a 3-profile solution would best fit the data. Subsequently, it was hypothesized that 2) children in the more optimal parenting practices class would report lower PTSS and fewer psychosocial difficulties while also endorsing more PTG and resilience compared to the other two classes, and 3) children in the more negative parenting practices class would report higher levels of PTSS and more psychosocial difficulties while also reporting less PTG and resilience compared to the other two classes. Analyses controlled for relevant covariates including children’s complicated grief symptoms, time since death, and relationship to the deceased.

## Method

### Participants

Participants included 112 children, aged 8 to 17 (*M*_*ag*e_ = 12.41, *SD* = 2.58; 52.2% female), who had experienced the death of a loved one within the past five years. The sample consisted primarily of White (46.0%) and Black (45.1%) children. Of the remaining participants, 6.2% identified as Biracial, 1.8% Asian, and 0.9% Native American; 8.0% identified as Latino or Hispanic. More than half of the participants had experienced the death of a parent (59.6%), 21.5% a grandparent, 10.8% a sibling, 7.2% an extended family member (e.g., aunt), and 0.09% a godparent. At the time of their interview, the average time since death was about 13 months (*M* = 12.84, *SD* = 11.26) and 89.38% had attended at least one grief-related service (e.g., grief camp, individual therapy, peer support group) since the death. Children answered parenting questions in relation to their primary caregiver who was responsible for their day-to-day care. Regarding children’s relationship to their caregiver, 69.0% answered parenting questions about their biological mother, 12.4% their biological father, 6.2% their grandmother, 6.2% their aunt, 2.7% their adoptive mother, 0.9% their adoptive father, 0.9% their stepmother, 0.9% their stepfather, and 0.9% their sister. Children came from families with highly variable socioeconomic status, with annual incomes ranging from <$40,000 (28.3%) to >$100,000 (23.0%) per year. Parental educational attainment also varied widely between families. Most participating caregivers had obtained at least a bachelor’s degree (63.8%), 21.3% had attended some college, 12.4% graduated high school, and 2.7% had attended some high school but did not graduate.

### Procedures

Institutional review board (IRB) approval for this study was obtained from Baptist Memorial Hospital System, with facilitated review from The University of Memphis. Following IRB approval, families were recruited through community organizations (primarily organizations serving bereaved individuals) from the MidSouth, United States. While most children had received some form of grief therapy services, some had not; enrollment in grief services was not a criterion for study inclusion. Potentially eligible families were screened by study staff. Children were eligible if they were between 8 and 17 years old (lower limit of eight given developmental understanding of the finality of death, Speece & Brent, 1984; upper limit of 17 because children are still minors and living with their primary caregivers), spoke English, lost a loved one within the past five years, and had a primary caregiver older than 18 who was willing to participate in the study. Prior to enrolling in the study, parents gave permission for their child to participate and children provided assent. Interviews took place at various community locations or in participants’ homes. Interviews were conducted by trained study staff and lasted between 60 and 90 minutes. Study staff read the survey questions aloud to all participants to increase comprehension. Children received a $15 gift card as compensation for their time, as well as a list of local and national mental health resources.

### Measures

#### Class Predictors

##### Parenting Practices

The Alabama Parenting Questionnaire (APQ) is a 42-item measure assessing the quality of the parent-child relationship across five subscales (Shelton et al., 1996). Two subscales represent positive parenting strategies: Parental Involvement (e.g., “You have a friendly talk with your caregiver”) and Positive Parenting Practices (e.g., “Your caregiver compliments you when you have done something well”). Three subscales represent negative parenting: Poor Monitoring/Supervision, Inconsistent Discipline and Corporal Punishment. The internal consistency of the Corporal Punishment scale is poor in past work (Grogan-Kaylor et al., 2020; Shelton et al., 1996), and psychometric studies suggest that the negative parenting items are best captured by two subscales, which were used in the current study: Ineffective Discipline (e.g., “Your caregiver threatens to punish you and then does not do it”) and Deficient Monitoring (e.g., “Your caregiver does not know the friends you are with”; Hinshaw et al., 2000). Response options range from 1 (*never happens*) to 5 (*always happens*), and items are summed to create total scores for Parental Involvement (α =.82), Positive Parenting (α =.83), Ineffective Discipline (α =.60), and Deficient Monitoring (α =.78).

##### Parent-Adolescent Communication

The Parent-Adolescent Communication Scale (PACS) uses 20-items to assess positive and negative aspects of family communication (Barnes & Olson, 2003). Children responded based on their typical communication with their caregiver. The PACS contains two subscales assessing Open Communication (e.g., “My caregiver is always a good listener”) and Problems in Communication (e.g., “I am afraid to ask my caregiver for what I want”). Response options range from 1 (*Strongly disagree*) to 5 (*Strongly agree*). Total subscale scores are created by summing the 10 items in each subscale. Past work demonstrates adequate internal consistency for both Open Communication (α =.87) and Problems in Communication (α =.78; Barnes & Olson, 2003). Internal consistency was also acceptable in the current study (α =.87 for Open Communication; α =.77 for Problems in Communication).

#### Study Outcomes

##### Posttraumatic Stress Symptoms

The 27-item symptom scale of the UCLA Posttraumatic Stress Disorder Reaction Index (PTSD-RI) was used to assess current posttraumatic stress symptom levels following an adverse life event (Steinberg et al., 2013). In line with measure instructions, children responded to the items based on their most traumatic life experience (MTE); 88.5% reported their MTE was the death of a loved one. Children rated the frequency of their PTSS over the past month from 0 (*None/Never*) to 4 (*Most/Almost every day*). A sample item is: “You try to stay away from people, places, or things that remind you about what happened.” A total score was calculated based on endorsed symptoms; higher scores reflect more PTSS. The total UCLA PTSD-RI symptom score shows adequate reliability in past work (α_range=_.67–.90; Steinberg et al., 2013). In this sample, Cronbach’s alpha was.89.

##### Psychosocial Difficulties

The Youth Self-Report (YSR) is a 112-item measure where children report on their behavioral, emotional, and social functioning over the past six months (Achenbach & Rescorla, 2001). Items are rated on a Likert scale from 0 (*Not True*) to 2 (*Very True/Often*). The syndrome scales assess children’s psychosocial difficulties including: Anxious/Depressed (e.g. “cries a lot”), Withdrawn/Depressed (e.g., “rather be alone”), Rule Breaking (e.g., “steals at home”), Somatic Complaints (e.g., “feels dizzy”), Aggression (e.g., “argues a lot”), Social Problems (e.g., “jealous of others”), Thought Problems (e.g., “harms self”), and Attention Problems (e.g., “acts young”). Computerized ASEBA scoring software was used to generate a T score and percentile across syndrome scales that accounted for child age and gender. Cronbach’s alpha was.92 for the total psychosocial difficulties score.

##### Posttraumatic Growth

The Posttraumatic Growth Inventory for Children-Revised (PTGI-C-R) is a 10-item scale measuring positive changes in children’s lives following a traumatic event (Kilmer et al., 2009). A sample item is: “I can now handle big problems better than I used to”. Response options range from 0 (*No Change*) to 3 (*A Lot of Change*) and are summed for a total score; higher scores represent more PTG. The PTGI-C-R shows good internal reliability in past studies (α =.77; Kilmer et al., 2009). In this sample, Cronbach’s alpha was.81.

##### Resilience

The Connor-Davidson Resilience Scale (CD-RISC) uses 25-items to assess one’s ability to cope with stress and adversity (Connor & Davidson, 2003). A sample item is: “I am not easily discouraged by failure.” Responses are rated on a Likert scale from 0 (*not true at all*) to 4 (*true nearly all of the time*) with higher scores indicating greater resilience. The CD-RISC displays good internal reliability (α =.89; Connor & Davidson, 2003) in previous work and in the current study (α =.87).

#### Covariates

##### Demographic and Loss-Related Variables

Children reported on their age and gender and answered loss-related questions including, “When did this death occur?” and “How did you know this person?” Time since the death was recorded in months. The child’s relationship to the deceased was coded into a dichotomous non-parental loss (0) and parental loss (1) variable.

##### Complicated Grief

The Inventory of Complicated Grief-Revised Child (ICG-RC) is a 28-item measure used to identify prolonged grief reactions (Melhem et al., 2013). Participants responded to items such as, “I feel that it is unfair that I should live when he/she has died.” Items are rated on a Likert scale from 1 (*Almost never/less than once a month*) to 5 (*Always/several times a day*) and are summed to create a total score. Higher scores represent more complicated grief symptoms. The ICG-RC has strong internal (α =.94) and test-retest reliability in past work (α =.80; Melhem et al., 2013). In this study, Cronbach’s alpha was.93.

### Data Analytic Plan

Data were screened for multivariate outliers using Mahalanobis’s distance, non-normality (VIF >2), skewness, and kurtosis in SPSS version 28 (IBM Corp, 2020). No problematic outliers were identified, and all independent variables met normality assumptions. Less than 1% of the data were missing and item-level mean imputation was used to address missingness. Continuous variable means, standard deviations, and correlations are presented in Table 1.

**Table 1:** Means, Standard Deviations, and Correlations Among Continuous Study Variables

	1	2	3	4	5	6	7	8	9	10	11	12	13
1. Child Age	–												
2. Time Since Death	−.01	–											
3. Complicated Grief	.07	.07	–										
4. Parental Involvement	−.19^*^	.22^*^	.08	–									
5. Positive Parenting	−.21^*^	.07	.09	.72^**^	–								
6. Deficient Monitoring	.41^**^	−.01	.09	−.29^**^	−.19*	–							
7. Ineffective Discipline	.17	.08	.20^*^	.01	.05	.57^**^	–						
8. Open Communication	−.19^*^	.07	.00	.62^**^	.55^**^	−.38^**^	−.18	–					
9. Problems in Communication	.32^**^	.10	.19^*^	−.29^**^	−.36^**^	−.46^**^	.45^**^	−.47^**^	–				
10. PTSS	.04	−.04	.76^**^	−.10	−.06	.01	.05	−.06	.14	–			
11. Psychosocial Difficulties	.20*	.42^**^	.42^**^	−.28^**^	−.28^**^	.41^**^	.44^**^	−.30^**^	.50^**^	.52^**^	–		
12. Posttraumatic Growth	.23	.27^**^	.27^**^	.03	.09	.12	.06	.12	.19*	.13	.09	–	
13. Resilience	.16	.08	.08	.31^**^	.27^**^	−.04	.05	.35^**^	−.10	.05	.29^**^	−.15	–
*M*	12.41	12.84	67.06	36.11	23.26	16.87	13.30	13.30	39.29	27.46	13.11	16.28	55.63
*SD*	2.58	11.26	22.17	7.36	4.86	6.42	4.00	4.00	7.54	7.54	10.91	6.88	10.60

*N* = 112. *p <.05. **p <.01; *M* = mean, *SD =* standard deviation; *Time since loss* = months since death of loved one, *PTSS* = posttraumatic stress symptoms

Prior to running the analysis, class predictors (i.e., Parental Involvement, Positive Parenting, Ineffective Discipline, Deficient Monitoring, Open Communication, Problems in Communication) were mean centered. The three-step approach to mixture modeling was used to examine study hypotheses using Mplus 8 (Muthén & Muthén, 2019). First, latent profiles were derived from the six parenting variables. The best profile solution for models 2 through 5 was determined based on several fit indices: the Bayesian Information Criterion (BIC; Schwarz, 1978), Akaike Information Criterion (AIC; Akaike, 1974), the lo-Mendell-rubin test (LMR; Lo et al., 2001), and the Bootstrap Likelihood Ratio test (BLRT; McLachlan & Peel, 2000). Model entropy above .80 indicated appropriate profile separation (Ramaswamy et al., 1993); profile solutions were also assessed for parsimony. The model with the best fit and most distinct profiles was selected. Next, the R3STEP estimation technique was used to assess the influence of the covariates (i.e., child age, complicated grief symptoms, time since death, relationship to the deceased) on profile membership (Asparouhov & Muthén, 2014). Finally, differences in children’s PTSS, psychosocial difficulties, PTG, and resilience were examined in relation to profile membership while controlling for significant covariates (Asparouhov & Muthén, 2014).

## Results

### Class Determination

Prior to selecting the best fitting model, fit criteria were independently reviewed by the first and second authors. Both researchers selected the 3-class model as the optimal solution based on its lower BIC value, significant BLRT, and distinctness of the three profiles (See Table 2). While the AIC decreased with each class addition, indicating that more profiles were preferable, the BIC value began increasing after the 3-class solution, suggesting that the 3-class solution was preferable compared to the other class solutions. In addition, the BLRT test reflected a significant improvement between the 2-class and 3-class solutions (BLRT = 81.25, *p* < .001), but not between the 3-class and 4-class models (BLRT = 60.45, *p* =.11). When comparing the 2-class and 3-class solutions, the 3-class solution yielded a meaningful third profile (e.g., Passive Parenting) that was not captured by the 2-class solution. Although the LMRT suggested the 4-class solution was a good fit for the data, the 4-class solution did not include a distinct fourth profile over the 3-class model. Finally, the fit indices for the 5-class solution did not demonstrate significant improvement over the 4-class model.

**Table 2:** Fit Statistics for Latent Class Solutions Two through Five

Number of Profiles	Loglikelihood	AIC	BIC	Entropy	LMR	LMR *p*	BLRT	BLRT *p*	*n* of smallest class
Indicator Variances Freed Across Profiles									
2	−851.74	1753.48	1821.44	0.93	194.38	<.001	197.55	<.001	33
**3**	**−811.11**	**1698.22**	**1801.53**	**0.87**	**79.95**	**0.64**	**81.25**	**<.001**	**25**
4	−780.89	1663.77	1802.41	0.90	59.48	0.02	60.45	0.11	13
5	−756.12	1640.24	1814.23	0.92	48.73	0.29	49.53	1.00	10

Class indicators were standardized prior to running the analyses; indicator variances were constrained across classes; *AIC* = Akaike Information Criterion, *BIC* = Bayesian Information Criterion, *LMR* = Lo-Mendell-Rubin Test, *LMR p* = Lo-Mendell-Rubin Test p value, *BLRT =* Bootstrap Likelihood Ratio Test, *BLRT p* = Bootstrap Likelihood Ratio Test p value. The bolded class was selected as the best fitting class solution

### Class Descriptions

**Figure 1:**
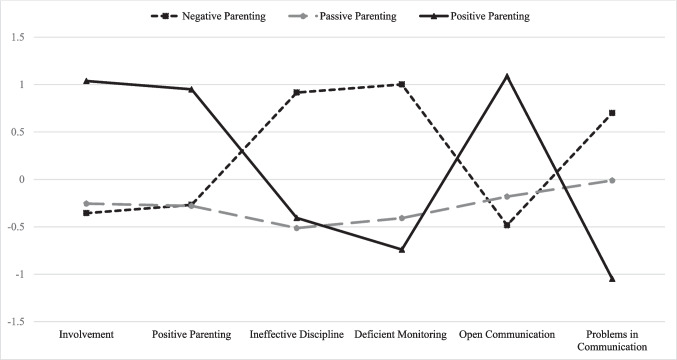
Three Class Solution of Parenting Practices *Note.* Negative Parenting class (*n* = 39, 34.82%); Passive Parenting class (*n* = 48, 42.86%); Positive Parenting class (*n* = 25, 22.32%)

In alignment with study hypotheses, three distinct parenting profiles yielded a good fit for the data. These profiles represented unique patterns of parenting practices among bereaved families. Figure 1 shows the 3-class solution, which includes a Passive Parenting class (*n* = 48, 42.86%), Negative Parenting class (*n* = 39, 34.82%), and Positive Parenting class (*n* = 25, 22.32%). The Passive Parenting class was the largest, while the Negative Parenting class represented about one-third of the sample; the Positive Parenting class was the smallest. In the Passive Parenting class, children endorsed low average levels of both positive and negative parenting practices used by their parents, which suggests that these children perceived their caregivers to be generally disengaged from parenting interactions. The Negative Parenting class was characterized by the highest reports of Deficient Monitoring, Ineffective Discipline, and Problems with Communication compared to the other two classes. Children in this class indicated that their parents were fairly uninvolved in their daily activities, used few positive reinforcement strategies, and had notable communication difficulties. In contrast, those in the Positive Parenting class reported their parents were actively involved in their lives, frequently used positive parenting practices, implemented appropriate supervision and effective discipline, and prioritized open communication.

### Predictors of Profile Membership

The R3STEP procedure (Asparouhov & Muthén, 2014) examined associations between covariates and profile membership. Relationship to the deceased predicted profile membership such that children in the Passive Parenting class were more likely to report that a parent died compared to those in the Positive Parenting Class (EST = 1.19, *p* = 0.032); neither child age, complicated grief symptoms, nor time since death were significantly associated with class membership. Time since death was related to children’s psychosocial difficulties (est = −0.15, p = .006), and children’s complicated grief symptoms were associated with their PTG (est =.08, *p* = .004), PTSS (est = .38, *p* <.001), and psychosocial difficulties (est =.16, *p* < .001); child age and relationship to the deceased were not associated with study outcomes. Given the impact on profile membership and/or study outcomes, time since death, complicated grief symptoms, and relationship to the deceased were retained as covariates.

### Associations between Parenting Profiles and Child Functioning

Wald’s tests, adjusted for covariates, were used to determine differences in PTSS, psychosocial difficulties, PTG, and resilience across parenting profiles (See Table 3). Cohen’s *d* was used to examine the magnitude of the effects between classes on each outcome (e.g., small effect = 0.20, medium effect = 0.50, or large = 0.80). As hypothesized, children in the Positive Parenting class reported significantly fewer psychosocial difficulties compared to those in the Passive [est = 8.54, SE = 2.18, *p* < .001; cohen’s *d* = 0.86] and Negative [est = 15.92, SE = 2.03, *p* < .001; cohen’s *d* = 1.67] Parenting classes; those in the Passive Parenting class also had fewer psychosocial difficulties than those in the Negative Parenting class [est = −7.38, SE = 1.87, *p* < .001; cohen’s *d* = 0.79]. In line with study hypotheses, children in the Positive Parenting class had significantly higher levels of resilience compared to both the Passive Parenting [est = −12.20, SE = 3.12, *p* < .001; cohen’s *d* = 0.69] and Negative Parenting [est = −9.30, SE = 3.76, *p* = .013; cohen’s *d* = 0.51] classes. Children in the Passive and Negative Parenting classes did not significantly differ on their reports of resilience. Contrary to what was hypothesized, no differences in PTSS or PTG emerged between any of the classes.

**Table 3:** Means and Class Comparisons for PTSS, Psychosocial Difficulties, Posttraumatic Growth, and Resilience

	Negative Parenting Class	Passive Parenting Class			Positive Parenting Class
**Distal Outcome**	**Mean (*SE*)**	**Mean (*SE*)**			**Mean (*SE*)**
PTSS	15.83 (1.91)	12.93 (1.69)			10.23 (1.99)
Psychosocial Difficulties	62.93 (1.33)	54.87 (1.40)			45.97 (1.84)
Posttraumatic Growth	16.79 (1.23)	16.54 (0.91)			15.03 (1.61)
Resilience	68.16 (2.63)	65.20 (1.90)			76.33 (2.40)
**Covariates**					
Complicated Grief Symptoms	71.78 (3.76)	66.10 (2.87)			61.78 (4.48)
Child Age	13.23	12.1			11.48
Child Gender	1.49	1.48			1.65
Parental Educational Attainment	15.13	15.80			16.09
Time Since Death	14.48	12.25			11.49
Relationship to Deceased	0.55	0.72			0.44
**Class Comparisons on Distal Outcomes**					
**PTSS**	**Estimate (SE)**	**Estimate/SE**	***p*** **value**	**CI**	**Cohen’s** ***d***
Negative vs. Passive	0.54 (1.88)	0.29	0.775	[−2.55–3.63]	0.22
Negative vs. Positive	2.13 (1.51)	1.41	0.160	[−0.36–4.62]	0.42
Passive vs. Positive	1.59 (1.52)	1.05	0.296	[−0.91–4.09]	0.20
**Psychosocial Difficulties**					
Negative vs. Passive	7.38 (1.87)	3.94	<0.001	[4.30–10.46]	0.79
Negative vs. Positive	15.92 (2.04)	7.81	<0.001	[12.57–19.27]	1.67
Passive vs. Positive	8.54 (2.18)	3.93	<0.001	[4.96–12.12]	0.86
**Posttraumatic Growth**					
Negative vs. Passive	−0.19 (1.58)	−0.12	0.905	[−2.81–2.42]	0.03
Negative vs. Positive	0.93 (2.03)	0.46	0.646	[−2.41–4.28]	0.21
Passive vs. Positive	1.12 (1.90)	0.59	0.554	[−2.00–4.24]	0.18
**Resilience**					
Negative vs. Passive	2.90 (3.41)	0.85	0.394	[−2.70–8.51]	0.18
Negative vs. Positive	−9.30 (3.76)	−2.47	0.013	[−15.48 - −3.11]	−0.51
Passive vs. Positive	−12.20 (3.32)	−3.68	<0.001	[−17.66 - −6.75]	−0.69

*N* = 112. *SE =* standard error, *PTSS =* posttraumatic stress symptoms; Parental Educational Attainment = number of years in school; Time Since Death = months since death of loved one; Relationship to Deceased = nonparental death compared to parental death; child gender coded as 1 = male, 2 = female; CI = confidence interval. All outcomes examined using covariate-adjusted estimates, controlling for complicated grief symptoms, time since death, and relationship to the deceased

## Discussion

There is an established link between the caregiving environment and children’s mental health outcomes following childhood adversity, such as the death of a loved one (Alvis et al., 2023; Wolchik et al., 2009). Although the parent-child relationship is nuanced and multifaceted, previous literature relies primarily on variable-centered approaches when quantifying the nature of this caregiving relationship (Howard Sharp et al., 2020; Morris et al., 2016). To advance the childhood bereavement literature, the current study used a person-centered approach to examine patterns of parenting practices in a sample of children who lost a loved one. Further, examining how these profiles are linked to both adaptive and maladaptive outcomes constitutes a study strength. Results revealed three patterns of parenting that represented caregivers who largely engage in positive parenting practices, those who largely engage in negative parenting practices, and those with a more passive parenting approach. Results also showed the benefits of a positive parent-child relationship for bereaved children’s psychosocial functioning, as indicated by low levels of psychosocial difficulties accompanied by high levels of resilience among children in the Positive Parenting class. Findings extend the field’s understanding of how children view their relationship with their parents, as well as how these views are associated with both maladaptive and adaptive outcomes in the context of a major loss.

Consistent with the first hypothesis, a three-class solution emerged as the best fitting model. The Positive Parenting class represented almost one-quarter of the sample, indicating that a sizeable portion of bereaved children perceive their relationship with their primary caregiver to be characterized by high levels of Parental Involvement, Positive Parenting practices, and Open Communication. This class aligns with previous studies of non-bereaved children, which have identified a positive parenting class characterized by high levels of positive parenting strategies and low levels of negative or harmful parenting strategies (Borden et al., 2014; Tzoumakis et al., 2015). The Negative Parenting class, which included about one-third of participants, showed that some bereaved children experience significant challenges in their relationship with their parents. This class is consistent with past work in other clinical populations demonstrating that some parents struggle to implement beneficial parenting strategies and may use more negative parenting techniques (e.g., inconsistent consequences, difficulties with communication; Borden et al., 2014; Tzoumakis et al., 2015). The emergence of the Passive Parenting class, representing the largest class, extends prior research by highlighting that many children felt that the parenting they received was not harmful but also not overtly helpful. While this class is supported by prior studies examining parenting profiles in which some parents take a more passive approach to parenting (e.g., Borden et al., 2014), it represented the largest class in the current study and suggests that the death of a loved one can have a significant impact on parenting practices. It is likely that parents are navigating many demands on their resources in the aftermath of a death and only have a limited capacity to engage with their children. For example, they may rely on others for regular supervision/monitoring of their child or reduce their involvement in their child’s activities to manage other responsibilities. Given that previous research was conducted with non-bereaved youth, the current study advances the literature by identifying this Passive Parenting profile in children who have experienced a death. Through the lens of the PDT framework (Mowder, 2005), results indicate that some parents adapt effectively to parenting in the context of bereavement, while others may struggle to adjust to their new circumstances.

Findings partially supported the second and third hypotheses. In terms of overall psychosocial difficulties, there is a clear link between parent-child interactions and children’s emotional and behavioral well-being. Children in the Positive Parenting class reported the most favorable psychosocial outcomes, followed by the Passive Parenting class, and then the Negative Parenting class. These findings align with research demonstrating associations between positive parenting and better mental health functioning in both bereaved and non-bereaved children (Bøe et al., 2014; Morris et al., 2016). Findings add to previous work by documenting differences in psychosocial difficulties between the Passive and Positive Parenting classes, which illustrates the unique value of positive parenting in reducing psychosocial challenges for children following the death of a loved one. While children in the Passive Parenting class reported fewer psychosocial difficulties than those in the Negative Parenting class, they comprised the largest class and had significantly more challenges than the Positive Parenting class. Results suggest that parents in the Passive Parenting class could perceive their children as “doing fine” and therefore not in need of more positive parenting behaviors; however, these children may benefit from increased communication, more parental involvement, and more positive reinforcement that could protect against current or future mental health challenges. Further, positive parenting practices had a unique connection to children’s resilience, such that children in the Positive Parenting class showed more resilience than those in the Negative or Passive Parenting classes. This finding supports past work demonstrating associations between positive parenting practices and children’s resilience (Cipriano et al., 2023). Taken together, these findings align with the RDS Metatheory, which suggests that bereaved children who experience appropriate support from their caregivers will be more equipped to adjust to the challenges they face in the context of significant loss (Lerner et al., 2018; Nader & Layne, 2009). Alternatively, perhaps children who display more resilience also reach out to their parents for support to navigate their grief. Additional research is needed to clarify directional relationships between parenting and bereaved children’s resilience.

Contrary to what was hypothesized, findings regarding trauma-specific outcomes yielded nonsignificant results, such that parenting profile membership was not associated with children’s PTSS or PTG. This finding was unexpected given past literature documenting links between parenting and PTSS (Afzal et al., 2023) and parenting and PTG (Howard Sharp et al., 2016) in samples exposed to other adversities. Some previous literature documents nonsignificant associations between parenting variables and PTSS (Williamson et al., 2017) as well as PTG (Kilmer & Gil-Rivas, 2010), which aligns with the current study. For example, a meta-analysis revealed that results regarding parenting and PTSS are highly variable across studies, and that parenting variables accounted for only 2.0–5.3% of the variance in children’s PTSS (Williamson et al., 2017). Thus, it is likely that other variables (e.g., genetic factors, parent mental health) play a role in children’s PTSS. Notably, children in this study reported on their parents’ general parenting behaviors and communication style, rather than bereavement-specific interactions. Perhaps assessing parent-child communication specifically about the death would have a more direct impact on mental health outcomes that are loss specific, such as children’s PTSS and PTG.

The exploration of covariates revealed that the child’s relationship to the deceased was associated with class membership. Children in the Positive Parenting class were less likely to report a parental loss compared to the Passive Parenting class, which could suggest that families in this class were experiencing less caregiving strain following the death. Perhaps children in the Positive Parenting class had parents with fewer grief or psychopathology symptoms, which made it easier for them to enact positive parenting practices. Such relations warrant study in future work, including how the loss of a spouse may impact parents’ perceptions of their parenting and their child’s adjustment. Further, time since death was related to study outcomes. Children who had experienced a more recent loss reported more psychosocial difficulties, a finding that likely reflects natural declines in bereavement-related distress over time (Haine et al., 2008). Complicated grief symptoms, which were also included as a covariate, were associated with higher levels of PTSS and psychosocial difficulties. These findings are consistent with previous studies demonstrating that bereaved children with more complicated grief also experience higher levels of PTSS (e.g., Spuij, Reitz, et al., 2012), internalizing problems, and externalizing problems (Spuij, Prinzie, et al., 2012). Curiously, time since death was not linked with parenting profile membership, which could suggest that parenting practices may be rather stable regardless of the timing of family adversity. Future longitudinal work is needed to explore how parenting patterns may change over time as well as before and after the death of a loved one. Complicated grief symptoms were also associated with higher levels of PTG, which aligns with prior work detailing how experiences of grief and PTG co-occur and interact with one another across time (Bellet et al., 2018). Indeed, the degree to which the loss is viewed as central to one’s identity and life story may predict grief and growth reactions (Bellet et al., 2018; Brookman et al., 2024).

### Clinical Implications

A variety of clinical implications emerge from these results. Regarding implications for treatment, findings underscore the value of family systems interventions given the relation between parenting factors and multiple aspects of bereaved children’s functioning. More specifically, children’s mental health and resilience may be bolstered by parent-child treatments that focus on enhancing positive parenting practices and open communication following a death. Interventions that support parents in the aftermath of a death may improve their capacity for positive parenting, which could lead to downstream benefits for their bereaved child’s well-being. These interventions may be particularly important for caregivers who have lost a spouse or co-parent. Regarding assessment implications, results showcase the value of using a person-centered approach to identify families at heightened risk for poor parenting and maladaptive child outcomes. The largest number of bereaved children were represented by the Passive Parenting class in which children perceived their caregivers to be generally disengaged. This passive approach to parenting was linked to more psychopathology and less resilience as compared to children in the Positive Parenting class; thus, children in both the Negative and Passive Parenting classes were experiencing psychosocial challenges and may benefit from treatment whereas children in the Positive Parenting class were functioning relatively well. As a proxy for identifying a dyad’s profile membership, therapists could directly assess parents’ enacted parenting behaviors during the intake interview with both the child and the parent. For example, general questions about the frequency of parenting behaviors (e.g., involvement, positive praise, monitoring, discipline, communication) could be incorporated into the intake assessment. Clinicians could also select specific items from the APQ or PACS to assess these parenting domains. The APQ has a 9-item short form (Elgar et al., 2007) that could be administered with relative ease in a clinical setting. These strategies may help therapists identify families most in need of services and assist with the development of a tailored treatment plan that addresses a family’s specific strengths and challenges.

### Limitations & Future Directions

The current study advances the childhood bereavement literature, but findings should be interpreted in light of several limitations. The sample size is small for the analytic approach that was utilized, even though it is quite large in comparison to other studies with bereaved children. The smaller sample may have impacted model power, prohibited the examination of moderating variables (e.g., child age, gender, time since death, relationship to the deceased), and limited the emergence of additional parenting profiles. Future researchers should aim to collect data from larger samples of bereaved children that could explore potential moderators between parenting profiles and child outcomes, including additional findings regarding children’s PTSS and PTG. The recruitment of a larger sample could also improve the internal consistency of some of the parenting subscales (i.e., Ineffective Discipline). Children in this study answered questions about PTSS in relation to their most traumatic experience; future researchers should consider asking participants to respond to PTSS items specifically in relation to the death of a loved one. The data are also cross-sectional, which prevents directional or causal claims regarding the relationship between parenting profiles and child functioning. Future work that collects data at multiple timepoints from several informants, including both child and parent reports of parenting, would allow for a longitudinal exploration of parenting and address bias incurred when using single-informant data. In addition, the longitudinal examination of parenting for families who specifically experienced the death of a parent would help provide a clearer picture of how surviving parents and children cope in the immediate aftermath of this loss, as well as over time. Further, the assessment of parent mental health especially following spousal loss should be included in future work. Future studies should also assess household composition (e.g., number of siblings, other family members in the home) as these variables could influence parent-child interactions. While the sample included a large number of Black children and children from a range of socioeconomic backgrounds, the generalizability of these findings is limited given that most children were recruited from grief centers; thus, future work should aim to include more children in the community who may not be engaging with services not specifically tied to bereavement. Finally, children’s complicated grief symptoms were included as a covariate to account for grief-specific symptoms across the sample as they could conflate the mental health variables explored in this work. Future research should explore how children’s complicated grief symptoms are influenced by parenting and if parents’ own grief symptoms mediate this association.

## Conclusion

Supportive parents play a vital role in facilitating children’s well-being in the aftermath of a death. This study employed a person-centered approach to identify patterns of positive and negative parenting behaviors. Results yielded three distinct parenting profiles including a Positive Parenting class, a Negative Parenting class, and a Passive Parenting class. Next, associations were explored between these classes and bereaved children’s maladaptive (i.e., PTSS, psychosocial difficulties) and adaptive (i.e., PTG, resilience) outcomes. Children in the Positive Parenting class had significantly fewer psychosocial difficulties and greater resilience compared to the other two classes. No differences were found across classes on PTSS or PTG. Findings can help tailor family-based interventions to bolster positive parent-child interactions, particularly among children with more disengaged parents, which could subsequently reduce children’s mental health challenges and build resilience following loss.

## Supplementary Information

ESM 1 (DOCX 21 kb)

## Data Availability

The study and research questions were not preregistered. Participant consents did not include permission for public release of deidentified data. As such, the data analyzed in this study are not available in a repository. Processed data for verification or meta-analyses are available from the first author by request. The MPLUS code for the current analyses has been uploaded as Supplement 1.
