# Supplementary material for: Patterns of Parenting and their Associations with Bereaved Children’s Maladaptive and Adaptive Functioning
Source: Res Child Adolesc Psychopathol. 2026 Feb 18;54(2):35. doi: 10.1007/s10802-025-01403-0 (PMC12917021; doi:10.1007/s10802-025-01403-0)
Supplement: Supplementary file 1 — (DOCX 21 kb) [file 10802_2025_1403_MOESM1_ESM.docx]

**Supplement 1 MPLUS Code**

**Class Determination**

VARIABLE:

NAMES ARE

ID

AGE

GENDER

RACE

ICG

EXTPROBT

INTPROBT

TOTPROBT

PTGI

PTSD

RELIGION

CYRM

CDRISC

ZINV

ZPOS

ZMON

ZINCN

CCP

ZCOR2

ZOC

ZPCR

TIME

EXPECTED

APQINCF

APQCORF

ZAPQINCF

ZAPQCORF

ZCCP

APQNEG

APQMON2

APQPOS2

ZNEG

ZMON2

ZAPOS2;

MISSING ARE ALL (-999);

USEVARIABLES ARE

ZINV ZPOS ZNEG ZMON2 ZOC ZPCR;

CLASSES = C (3);

ANALYSIS:

TYPE = MIXTURE;

STARTS = 4000 800; STITERATIONS = 800;

LRTSTARTS = 0 0 4000 1000; ! USE FOR BLRT

PROCESSORS = 8;

MODEL:

%OVERALL%

[ZINV ZPOS ZNEG ZMON2 ZOC ZPCR];

ZINV ZPOS ZNEG ZMON2 ZOC ZPCR;

%C#1%

ZINV ZPOS ZNEG ZMON2 ZOC ZPCR;

%C#2%

ZINV ZPOS ZNEG ZMON2 ZOC ZPCR;

%C#3%

ZINV ZPOS ZNEG ZMON2 ZOC ZPCR;

PLOT: TYPE=PLOT3; SERIES IS ZINV ZPOS ZNEG ZMON2 ZOC ZPCR(*);

OUTPUT: TECH7 ENTROPY TECH11 TECH14 SAMPSTAT PATTERNS;

**R3STEP Procedure**

VARIABLE:

NAMES ARE

ID

GENDER

RACE

EXTPROBT

INTPROBT

RELIGION

CYRM

ZINV

ZPOS

ZMON

ZINCN

CCP

ZCOR2

ZOC

ZPCR

EXPECTED

APQINCF

APQCORF

ZAPQINCF

ZAPQCORF

ZCCP

APQPOS2

ZNEG

ZMON2

ZAPOS2

AGE

TIME

ICG

INV

POS

APQMON2

APQNEG

PACSOC

PACSPCR

TOTPROBT

PTGI

PTSD

CDRISC

CHILDRELA

T1CLOSS4

PARENTLOSS

CLOSSRELA;

MISSING ARE ALL (-999);

USEVARIABLES ARE

ZINV ZPOS ZNEG ZMON2 ZOC ZPCR;

CLASSES = C (3);

AUXI = (R3STEP) ICG TIME EXPECTED PARENTLOSS AGE;

**ANALYSIS:**

TYPE = MIXTURE;

STARTS = 4000 800; STITERATIONS = 800;

LRTSTARTS = 0 0 4000 1000;

PROCESSORS = 8;

**MODEL:**

%OVERALL%

[ZINV ZPOS ZNEG ZMON2 ZOC ZPCR];

ZINV ZPOS ZNEG ZMON2 ZOC ZPCR;

%C#1%

ZINV ZPOS ZNEG ZMON2 ZOC ZPCR;

%C#2%

ZINV ZPOS ZNEG ZMON2 ZOC ZPCR;

%C#3%

ZINV ZPOS ZNEG ZMON2 ZOC ZPCR;

PLOT: TYPE=PLOT3; SERIES IS ZINV ZPOS ZNEG ZMON2 ZOC ZPCR(*);

OUTPUT: TECH7 ENTROPY TECH11 TECH14 SAMPSTAT PATTERNS;

**Wald’s Tests**

***BCH Step One***

VARIABLE:

NAMES ARE

ID

GENDER

RACE

EXTPROBT

INTPROBT

RELIGION

CYRM

ZINV

ZPOS

ZMON

ZINCN

CCP

ZCOR2

ZOC

ZPCR

EXPECTED

APQINCF

APQCORF

ZAPQINCF

ZAPQCORF

ZCCP

APQPOS2

ZNEG

ZMON2

ZAPOS2

AGE

TIME

ICG

INV

POS

APQMON2

APQNEG

PACSOC

PACSPCR

TOTPROBT

PTGI

PTSD

CDRISC

CHILDRELA

T1CLOSS4

PARENTLOSS

CLOSSRELA;

MISSING ARE ALL (-999);

USEVARIABLES ARE

ZINV ZPOS ZNEG ZMON2 ZOC ZPCR;

CLASSES = C (3);

AUXI = PTGI PTSD TOTPROBT CDRISC

ICG TIME PARENTLOSS ;

ANALYSIS:

TYPE = MIXTURE;

STARTS = 4000 800; STITERATIONS = 800;

LRTSTARTS = 0 0 4000 1000;

PROCESSORS = 8;

MODEL:

%OVERALL%

[ZINV ZPOS ZNEG ZMON2 ZOC ZPCR];

ZINV ZPOS ZNEG ZMON2 ZOC ZPCR;

%C#1%

ZINV ZPOS ZNEG ZMON2 ZOC ZPCR;

%C#2%

ZINV ZPOS ZNEG ZMON2 ZOC ZPCR;

%C#3%

ZINV ZPOS ZNEG ZMON2 ZOC ZPCR;

SAVEDATA: FILE IS D:\3CLASSPARENTALLOSSBCHSTEP1.DAT;

MISSFLAG=-999;

SAVE=BCHWEIGHTS;

***BCH Step Two***

VARIABLE:

NAMES ARE

ZINV

ZPOS

ZNEG

ZMON2

ZOC

ZPCR

PTGI

PTSD

TOTPROBT

CDRISC

ICG

TIME

PARENTLO

BCHW1

BCHW2

BCHW3;

USEVARIABLES ARE

PTGI

PTSD

TOTPROBT

CDRISC

ICG

TIME

PARENTLO

BCHW1

BCHW2

BCHW3;

MISSING ARE ALL (-999);

CLASSES = c (3);

Training= BCHW1

BCHW2

BCHW3 (bch);

ANALYSIS:

TYPE = MIXTURE;

STARTS = 0;

Estimator=mlr;

MODEL:

%OVERALL%

C on ICG TIME PARENTLO;

PTGI on ICG TIME PARENTLO;

PTSD on ICG TIME PARENTLO;

TOTPROBT on ICG TIME PARENTLO;

CDRISC on ICG TIME PARENTLO;

%C#1%

[PTGI](a1);

[PTSD](a2);

[TOTPROBT](a3);

[CDRISC](a4);

%C#2%

[PTGI](b1);

[PTSD](b2);

[TOTPROBT](b3);

[CDRISC](b4);

%C#3%

[PTGI](c1);

[PTSD](c2);

[TOTPROBT](C3);

[CDRISC](c4);

MODEL CONSTRAINT:

NEW (ab1-ab4 ac1-ac4 bc1-bc4);

DO (1,4) ab# = a#-b#;

DO (1,4) ac# = a#-c#;

DO (1,4) bc# = b#-c#;

OUTPUT: tech1 tech7 ENTROPY TECH4

SVALUES SAMPSTAT cinterval;
